# Microcephaly

**DOI:** 10.3390/children4060047

**Published:** 2017-06-09

**Authors:** Emily Hanzlik, Joseph Gigante

**Affiliations:** Department of Pediatrics, Vanderbilt University School of Medicine, 8161 Doctors’ Office Tower, 2200 Children’s Way, Nashville, TN 37232, USA; emily.hanzlik@vanderbilt.edu

**Keywords:** head circumference, microcephaly, syndromes, genetic abnormalities, neuroimaging, Zika virus

## Abstract

Microcephaly is defined as a head circumference more than two standard deviations below the mean for gender and age. Congenital microcephaly is present at birth, whereas postnatal microcephaly occurs later in life. Genetic abnormalities, syndromes, metabolic disorders, teratogens, infections, prenatal, perinatal, and postnatal injuries can cause both congenital and postnatal microcephaly. Evaluation of patients with microcephaly begins with a thorough history and physical examination. In cases of worsening microcephaly or neurological signs or symptoms, neuroimaging, metabolic, or genetic testing should be strongly considered. Any further studies and workup should be directed by the presence of signs or symptoms pointing to an underlying diagnosis and are usually used as confirmatory testing for certain conditions. Neuroimaging with magnetic resonance imaging (MRI) is often the first diagnostic test in evaluating children with microcephaly. Genetic testing is becoming more common and is often the next step following neuroimaging when there is no specific evidence in the history or physical examination suggesting a diagnosis. Microcephaly is a lifelong condition with no known cure. The prognosis is usually worse for children who experienced an intrauterine infection or have a chromosomal or metabolic abnormality. Zika virus has rapidly spread since 2015, and maternal infection with this virus is associated with microcephaly and other serious brain abnormalities. Microcephaly has become much more prevalent in the news and scientific community with the recent emergence of Zika virus as a cause of congenital microcephaly.

## 1. Definition

**Figure 1:**
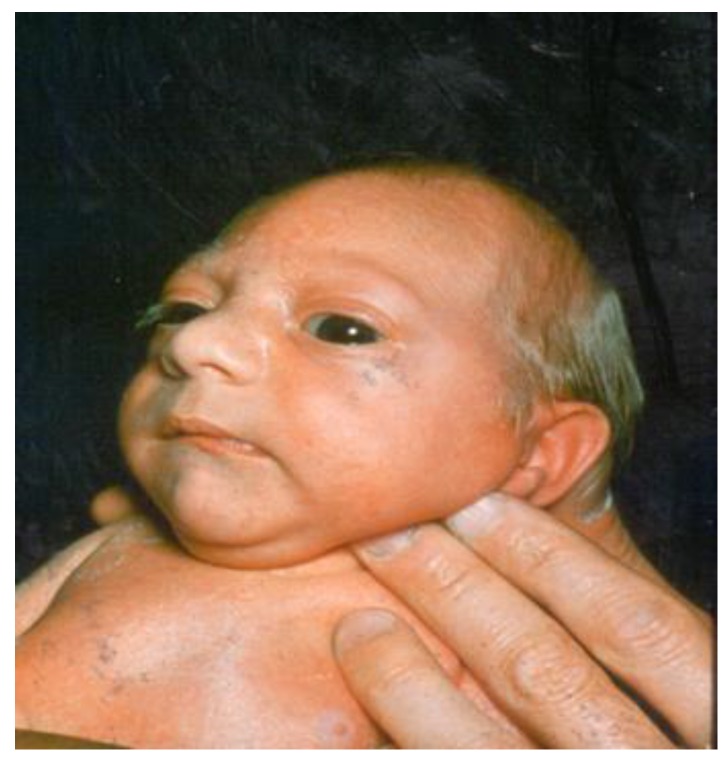
Newborn Infant with congenital microcephaly.

Microcephaly is a condition defined as a small head circumference present at birth (congenital microcephaly, Figure 1) or later in life (postnatal or acquired microcephaly). Children with postnatal microcephaly have a normal head size at birth then subsequently fail to have normal head growth. Microcephaly is defined as a head circumference more than two standard deviations (SD) below the mean for gender and age, which includes about 2% of the population [1,2,3,4,5,6,7]. Other studies define severe microcephaly as greater than three SD below the mean, which includes about 0.1% of the population [1,8,9,10]. Underreporting and varying study definitions make it difficult to track and report a true incidence of microcephaly. Another difficulty in using standard deviations to report, study, and monitor microcephaly is the concept of proportional microcephaly. Proportional microcephaly occurs when the head circumference is >2–3 SD below the mean in addition to height and weight being at similar percentiles. While low birth weight and proportional microcephaly has its own set of complications and prognosis, these children do not have the same prognosis and trends of neurocognitive outcomes as those with microcephaly in the setting of normal weight and height. Studies do not consistently separate the two, making information about prognosis more difficult to determine as well. The Center for Disease Control and Prevention (CDC) collects birth defects data including microcephaly and estimates the incidence to be 2–12/10,000 live births in the United States [11].

Syndromes, genetic abnormalities, metabolic disorders, infections, teratogens, prenatal, perinatal, and postnatal injuries can cause both congenital and postnatal microcephaly [1,4,7,10,12]. Microcephaly can also be seen as an isolated finding. Outcomes and prognosis vary based on the type (congenital vs. postnatal) and cause of microcephaly. However, without universally accepted standards in defining and reporting microcephaly, studies vary, and some authors separate etiologies and outcomes while others keep them combined, making predicting prognosis for affected patients difficult [4,10,11].

## 2. Etiology

Microcephaly has been stratified and studied differently based on the etiology and the timing of onset. For example, microcephaly can be stratified into genetic causes, those associated with syndromes or a known constellation of symptoms, secondary to insults to neuronal development including toxins, metabolites, and infections, and proportional microcephaly. Studies have shown that abnormal neuronal development and migration involving many genes may lead to microcephaly. Autosomal recessive primary microcephaly (MCPH) is associated with single gene mutations resulting in isolated microcephaly with often normal magnetic resonance imaging (MRIs) and no other physical examination findings. The current incidence ranges from 1.3–150/100,000 depending on the level of consanguinity of the population [13]. There continues to be more genes and novel mutations being discovered [13,14,15,16,17,18,19]. As of December 2016, 17 genes (*MCPH1-17*) have been identified that are associated with genetic causes of congenital microcephaly. The mutations involved are most often premature stop codons resulting in the halt of cell cycle progression, causing centromere abnormalities that lead to early apoptosis [14]. The discovery of these genes and mutations have helped guide the understanding of other etiologies of microcephaly such as infections, toxins, or teratogens by their mechanism of action on cell proliferation. As exome sequencing becomes more readily available and interpretable, more genes linked to microcephaly are expected to be discovered [7,9]. There are over 800 known syndromes with microcephaly as a known association and over 900 Online Mendelian Inheritance in Man (OMIM) conditions linked to microcephaly [1,10,20]. Congenital microcephaly can also result from insults during pregnancy that stop the brain from growing and developing normally. Environmental insults including hypoxic injury, maternal metabolic abnormalities such as Phenylketonuria (PKU), teratogen exposure, and infections can interfere with brain development and lead to congenital microcephaly [1]. Congenital infections causing microcephaly are known as TORCH infections (Toxoplasmosis, Other infections (Syphilis, Varicella Zoster, Parvovirus B-19), Rubella, Cytomegalovirus (CMV) and Herpes virus). In addition to microcephaly, children with these intrauterine infections often have other abnormal clinical findings which can include hepatosplenomegaly, rashes, chorioretinitis, and intracranial calcifications. Recently a rise in the number of prenatal Zika virus infections has been linked with microcephaly and other serious brain abnormalities.

While the majority of cases of microcephaly are congenital, there are cases where insults occur postnatally that affect brain development. Postnatal microcephaly can be genetic and present with normal head circumference at birth, but underlying genetic predisposition results in the failure of proper brain growth (e.g., Rett syndrome). Acquired microcephaly can also be due to many of the same insults that may occur during pregnancy, including cerebrovascular incidents, hypoxia, metabolic derangements, or infections. A retrospective study of a cohort of 680 children with microcephaly reported a known etiology in 59% of all patients. In those with a known etiology, about half were due to a genetic cause, 45% were associated with perinatal injury, and 3% were caused by postnatal injury [5]. Determining the etiology of microcephaly can help further research into the prevention of this condition. It can also provide means for predicting a prognosis and planning early interventions that can aid patients and their families.

## 3. Evaluation

**Figure 2:**
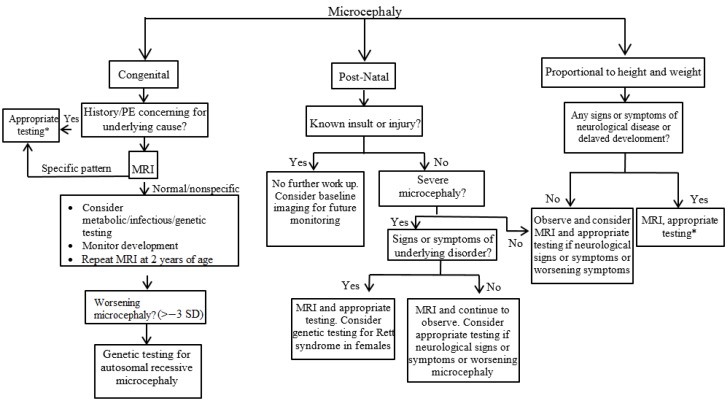
Diagnostic approach to a child with microcephaly. *Testing for infections, genetic, metabolic, toxin, and endocrine disorders. MRI: magnetic resonance imaging; SD: Standard Deviation.

Diagnosis and evaluation of microcephaly is warranted to determine the underlying cause, potential associated conditions, and aid with prognosis. A thorough history and complete physical examination are the first steps in evaluating a child with microcephaly. Important parts of the history include a detailed prenatal history, with specific questions about maternal medical problems, infections, medications and substance use (e.g., alcohol, tobacco, illicit drugs). Perinatal complications and infections at the time of birth can cause microcephaly. History should also include the age at onset, severity, and family history to determine if there are other similarly affected family members, consanguinity, or metabolic and genetic diseases. Measuring parental head circumferences is important, as it may help diagnose familial microcephaly. With regard to the physical examination, dysmorphic facial features and other abnormalities may suggest a diagnosis or direction for further testing. Head circumference should be measured and compared to previous measurements. Diagnostic approaches to congenital, postnatal, and proportional microcephaly are proposed in Figure 2.

The American Academy of Neurology has published practice parameters in the evaluation of children with microcephaly [3]. If the microcephaly is proportionate to height and weight and the child has no neurologic signs or symptoms or family history of neurologic disease, observation is recommended with no further studies apart from a close follow-up of development. MRI, genetic, or metabolic testing should be considered if the child develops neurologic signs or symptoms or worsening microcephaly. In cases where the microcephaly is not proportionate or the diagnosis is not clear following a thorough history and physical examination, neuroimaging should be considered as the next step. Computed tomography (CT) of the head is often non-specific but does have a strong prognostic value if abnormalities are noted. MRI is often performed early in infancy but is typically not urgently needed. MRI is more sensitive than CT and is therefore the gold standard for imaging in evaluation of the etiology of microcephaly. Even if MRI performed early in infancy is normal, repeated MRI after two years of age is recommended given complete myelination at this age [5]. In those with severe microcephaly (greater than three SD below the mean), abnormal findings on MRI were more likely than those with less severe microcephaly (80% vs. 43%) [21]. Another study evaluating those who developed postnatal microcephaly found that 100% had abnormal MRI findings, usually hydranencephaly or infarction [22].

After neuroimaging, there is no universal testing recommended. Any further studies and workup should be directed by the presence of signs or symptoms pointing to an underlying diagnosis and are usually used as confirmatory testing for certain conditions. These studies may include specific metabolic testing when a strong clinical suspicion based on the physical exam or family history is suspected. Isolated microcephaly caused by metabolic disorders with no other signs is rare, with three noted exceptions: maternal PKU, Amish lethal microcephaly, and phosphoglycerate dehydrogenase deficiency. In patients with microcephaly and global developmental delay, the prevalence of an underlying metabolic disorder ranges from 1–5% but evidence is limited due to the rare nature of most metabolic disorders [23]. Further studies with electroencephalogram (EEG) and ophthalmology referral are only recommended with clinical signs or concerns. While routine EEGs are not recommended, parents of children with microcephaly should be given information on seizures and precautions due to the increased incidence seen in those with microcephaly [3].

Genetic testing is the new frontier in evaluating the etiology of microcephaly as both specific genetic testing and whole exome sequencing become more readily available. Studies have shown that genetic testing and chromosomal microarrays can determine an underlying cause of microcephaly in 15.3–52% of cases [24,25]. Currently, there is not enough evidence to support genetic testing in the evaluation of all cases of microcephaly, but it is often the next step following neuroimaging when there is no specific evidence in the history or physical examination suggesting a diagnosis [3,5]. Whole exome sequencing is becoming more widely available and is being studied in the evaluation of microcephaly. Use of this technology found an etiology in 29% of previously evaluated and undiagnosed cases of microcephaly [9].

## 4. Prognosis

Microcephaly is a lifelong condition with no known cure. The prognosis is usually worse for children who experienced an intrauterine infection or have a chromosomal or metabolic abnormality. Depending on the cause and severity, children with microcephaly can have a number of different problems. These include intellectual disability, developmental delay, epilepsy, cerebral palsy, as well as ophthalmologic and audiologic disorders. A retrospective study of 680 children reported that 65% of children with microcephaly had intellectual impairment, 43% had epilepsy, and 30% had ophthalmological disorders [5].

## 5. Specific Focus on Congenital Infections and Microcephaly: the Emergence of Zika Virus

Microcephaly has become prevalent in the news and medical community with the emergence of Zika virus and the associated severe microcephaly with infection during pregnancy. Prior to Zika virus, the most common congenital infections causing microcephaly were TORCH infections. Cytomegalovirus is the most recognized and common TORCH infection causing microcephaly [26,27]. Cytomegalovirus, when symptomatic, has a 20% microcephaly rate, which is the strongest predictive value for future neurocognitive deficits. In congenital CMV, head circumference at birth shows a high correlation to intelligence quotient (IQ) scores in childhood [21]. Rubella and varicella are also known congenital infections causing microcephaly and intellectual deficits but are less common following the implementation of rubella and varicella vaccines. While vaccines have significantly decreased the rates of microcephaly secondary to congenital rubella syndrome, the emergence of non-vaccinated persons in developed countries has the potential to increase the rates of congenital rubella syndrome and microcephaly. Microcephaly due to Zika virus is a new epidemic, and long-term outcomes are unknown. In addition to microcephaly, other neurologic abnormalities have been observed in infants with congenital Zika virus infections including intracranial calcifications (mostly periventricular), hearing loss, ocular findings (chorioretinal atrophy, optic nerve atrophy, abnormal retinal vasculature), ventriculomegaly, polymicrogyria, hypoplasia of the cerebellum, and corpus callosum [28]. In the future, Zika virus may become a new common TORCH infection.

Microcephaly and abnormal brain development from other etiologies have been studied and can be used to help with future prognosis in those with Zika virus microcephaly. Outcomes are highly variable and depend on the underlying cause. Isolated and idiopathic/familial microcephaly have not been associated with decreased developmental outcomes or IQ scores when no other deficits are noted [4,5]. One study that followed children’s head circumference growth parameters prenatally through childhood found no correlation between neurocognitive outcomes and head circumference alone [2]. However, other studies looking at outcomes in severe microcephaly and those with known underlying causes and/or exposures have shown lower neurocognitive outcomes and motor findings. In one study looking at neuroimaging, MRI abnormalities in the setting of microcephaly showed correlation with lower Bayley Developmental Scores later in childhood [6]. Since the involvement of the CDC, World Health Organization (WHO), and the emergence of more cases of congenital Zika virus infections and microcephaly, extensive research in the mechanism of microcephaly has been explored. In vitro studies of Zika virus have shown that infections lead to apoptosis and death of neuronal progenitor cells (NPC) [28]. The virus invades NPCs and leads to abnormalities in cell division as well as the disruption of the centromeres [29,30]. This is a similar mechanism known to be disrupted in *MCPH* genes in autosomal recessive microcephaly as well as in radiation induced microcephaly [13,31]. In addition to congenital microcephaly, studies have also shown a mechanism in which Zika virus could contribute to postnatal head growth, independent of the time of infection (congenital vs. postnatal). In young infants, postnatal head growth could be affected based on evidence showing that Zika virus disrupts the vascular development of the blood brain barrier leading to exaggerated immune response, and gliosis leading to brain cell damage [32]. While outcomes in microcephaly related to Zika virus are unknown, the known association with decreased developmental scores and IQ in cases of other infections and teratogens, as well as the emerging understanding of the mechanism of neuronal involvement from Zika virus, will likely lead to recommendations of close neurodevelopmental follow-up of children known to be affected.

In 2015–2016, reports emerged from Brazil about a significant rise in the rates of microcephaly, up to 4–8% of all live births. The rise in Zika infections (transmitted by mosquitos) in Brazil during pregnancy became the leading hypothesis for the dramatic increase in microcephaly cases [33]. In April 2016, the CDC released a statement that, after extensive review of the evidence and literature and using criteria to establish causality when evaluating teratogens, it had concluded that a causal relationship exists between prenatal Zika virus infection and microcephaly and other serious brain defects [34]. The declaration of causality has allowed for more the direct involvement of the CDC and WHO in identifying and reporting cases. Additionally, increasing resources are being made available for the prevention and research of Zika virus including mosquito prevention and identification, and limiting travel to highly endemic areas. With the emergence of Zika virus and expected poorer outcomes associated with severe microcephaly, focusing on prevention and vaccine development to help decrease cases of congenital microcephaly are at the forefront of worldwide health agencies. As of February 2017, there is evidence of an effective low dose single time vaccine with protection from Zika virus in Rhesus monkeys, with further vaccine research ongoing [35].

## 6. Conclusions

Microcephaly is defined as a head circumference more than two standard deviations below the mean for gender and age. It may be present at birth or develop postnatally. While a number of genetic, metabolic, environmental, and infectious insults can disturb brain growth and cause microcephaly, no etiology is identified in up to 40% of cases. A thorough and detailed history and physical examination may suggest a diagnosis or further testing. Further diagnostic evaluation may include neuroimaging, and metabolic or genetic testing. Prognosis depends on the underlying cause of microcephaly. New genetic tests may provide novel diagnostic tools in the assessment of these patients. The recent rapid spread of Zika virus will lead to an increase in prenatal Zika virus infections, which will cause an increase in microcephaly and other serious brain abnormalities.
